# Kinetics of Surface Wettability of Aromatic Polymers (PET, PS, PEEK, and PPS) upon Treatment with Neutral Oxygen Atoms from Non-Equilibrium Oxygen Plasma

**DOI:** 10.3390/polym16101381

**Published:** 2024-05-12

**Authors:** Alenka Vesel, Rok Zaplotnik, Gregor Primc, Miran Mozetič

**Affiliations:** Jozef Stefan Institute, Department of Surface Engineering, Jamova cesta 39, 1000 Ljubljana, Slovenia

**Keywords:** aromatic polymers, wettability, water contact angle, atomic oxygen, flux, fluence

## Abstract

The wettability of polymers is usually inadequate to ensure the appropriate spreading of polar liquids and thus enable the required adhesion of coatings. A standard ecologically benign method for increasing the polymer wettability is a brief treatment with a non-equilibrium plasma rich in reactive oxygen species and predominantly neutral oxygen atoms in the ground electronic state. The evolution of the surface wettability of selected aromatic polymers was investigated by water droplet contact angles deposited immediately after exposing polymer samples to fluxes of oxygen atoms between 3 × 10^20^ and 1 × 10^23^ m^−2^s^−1^. The treatment time varied between 0.01 and 1000 s. The wettability evolution versus the O-atom fluence for all aromatic polymers followed similar behavior regardless of the flux of O atoms or the type of polymer. In the range of fluences between approximately 5 × 10^20^ and 5 × 10^23^ m^−2^, the water contact angle decreased exponentially with increasing fluence and dropped to 1/e of the initial value after receiving the fluence close to 5 × 10^22^ m^−2^.

## 1. Introduction

Surface activation of materials by treatment with oxygen plasma is a mature technology for improving polymer wettability [1]. The precise control of surface wettability is very important in various areas, such as biomedicine, energy, and environmental applications, as explained in recent review papers [2,3]. In gaseous plasma, oxygen molecules are partially excited to electronic and vibrational states, dissociated, and ionized [4]. The oxygen species of rather high potential energy interact with the surface of polymers, causing functionalization with oxygen-containing functional groups. The reactive oxygen plasma species include atoms in both the ground and metastable excited states, molecules in metastable excited states, and positively charged molecular and atomic ions. Furthermore, the positively charged ions are accelerated toward the surface in the electric field within the sheath between the bulk plasma and the surface and cause a weak bombardment of the polymer samples. Unless thin polymer samples are backed by an electrode powered by a high-frequency voltage, the kinetic energy of positively charged ions upon impinging the surface is several eV. The positive ions thus bring additional energy that is useful for the enrichment of surface chemical reactions. Plasma is also a source of radiation that often peaks in the vacuum ultraviolet (VUV) range, with photon energy between approximately 6 and 12 eV [5]. The VUV photons break bonds in the surface film and thus add to the complexity of the interaction between the oxygen plasma and the polymer surface [5,6].

The polar oxygen surface functional groups cause an increase in the polar component of the surface free energy and thus increased wettability [7]. The wettability is often measured by placing a small droplet of water on the polymer surface and measuring the contact angle (WCA). Many polymers are moderately hydrophobic, with the WCA between approximately 60 and 100° [8,9]. Such a rather high WCA may represent a drawback in any attempt to adhere a coating to the polymer substrate, so polymers are treated with oxygen plasma to ensure polar surface functional groups and, thus, adequate wettability.

Surface wettability obviously depends on the fluxes and fluences of various plasma species that are capable of interacting chemically with the polymer surface. Little surface modification will occur if the fluence is very low. If the fluence is very large, the polymer surface will heat significantly because all the surface reactions are highly exothermic, and the dissipated energy is likely to heat the polymer samples. The exothermic reactions include the dissipation of the positive ion kinetic energy to excite phonon states, the neutralization of charged particles, the relaxation of metastables, and the surface association of O atoms with parent molecules. Excessive heating will cause rapid surface hydrophobic recovery [10]. Between the two extremes (insufficient fluence to cause significant wettability and too much fluence, which causes excessive heating), there is a range of optimal fluence.

Interestingly enough, only a few authors reported the fluence of reactive species from the plasma when describing the evolution of the wettability of polymer samples. In fact, the majority of authors do not mention the fluences or fluxes at all. Instead, they report the surface wettability as a function of indirect parameters, such as the treatment time, the configuration of the experimental setup, the type of discharge, the gas pressure, flow, etc. The reported surface wettability for the same type of polymer thus varies significantly, depending on the specificity of the experimental conditions. Recent reviews indicate large discrepancies ranging from marginally improved wettability to super-hydrophilic surface finish at the same treatment times and for the same polymers [11]. One of the main reasons for such large discrepancies in wettability is the different fluences of reactive species as a consequence of different discharge configurations and different experimental conditions adopted by various research groups that have tackled this topic. The discrepancies between the results reported by different research teams may also arise from the synergetic effects of neutral reactive species, charged particles, and VUV photons.

**Figure 1:**
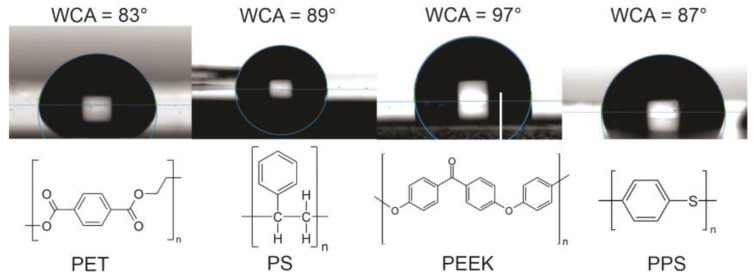
The structural formulae of selected aromatic polymers and the water contact angles of selected pristine samples.

In this paper, we focus on frequently used aromatic polymers such as polyethylene terephthalate (PET), polystyrene (PS), polyether ether ketone (PEEK), and polyphenylene sulfide (PPS). The structural formulae of these polymers are shown in Figure 1. The selected polymers differ in their chemical composition—PS is a pure hydrocarbon polymer, PET and PEEK contain oxygen, and PPS contains sulfur. The measured values of WCA for these non-treated polymers are also stated in Figure 1. The values reported in the literature by specific authors may differ by 10° or even more, which could be explained either by the accuracy of the device used for measuring the water contact angle, any surface impurities, different surface morphology, purity, and crystallinity, or a combination of these effects. We measured the WCA for all four aromatic polymers, and the water droplets for untreated polymer samples used in this study are shown in Figure 1.

The state-of-the-art evolution of the surface wettability of selected aromatic polymers treated with oxygen plasma or their flowing afterglow is summarized in Table 1. We present the initial WCA before plasma treatment, the lowest reported WCA after plasma treatment, the time needed to achieve maximum wettability, and also the basic properties of the experimental setups reported by various authors, including the type of discharge, the reported discharge power or power density, the gas pressure, and flow. The brief literature survey summarized in Table 1 indicates a large scattering of the wettability of all four aromatic polymers (PET, PS, PEEK, and PPS). Some authors reported excellent wettability already at several seconds of plasma treatment, whereas others obtained only moderate wettability despite treating polymer samples for several minutes. The results of the different authors are not comparable because they used various experimental configurations and have not reported the fluxes or fluences of reactive oxygen species. The large scattering of the WCA on plasma-treated polymers is, therefore, likely to be a consequence of huge differences in densities of plasma species in the plasma reactor and, thus, the fluxes and fluences of said species onto the polymer surfaces. Other reasons for the scattering of the reported results may be small differences in the polymer structure, like different molecular weights, degrees of orientation, and crystallinity, as well as additives used in the synthesizing procedure.

To clarify the wettability evolution of the above-mentioned aromatic polymers and to show the importance of the fluence of neutral oxygen atoms, we treated all polymer samples in the same experimental system and precisely dosed the oxygen atoms in the reaction chamber to achieve appropriate fluences at different fluxes of O atoms on the polymer surfaces.

## 2. Materials and Methods

### 2.1. Materials

The polymer samples were purchased from Goodfellow Ltd. (Huntingdon, UK). The following polymer foils were used: biaxially oriented polyethylene terephthalate (PET) with a thickness of 0.25 mm, biaxially oriented polystyrene (PS) with a thickness of 0.125 mm, amorphous polyether ether ketone (PEEK) with a thickness of 0.2 mm, and polyphenylene sulfide (PPS) with a thickness of 0.16 mm. The polymers were cut into small samples with a size of 1 cm × 3 cm. No pre-treatment was performed because the samples were taken directly from the sealed packages. Each sample was taped to a microscope slide before it was placed in the treatment chamber of our experimental system.

### 2.2. Treatment Procedure

**Figure 2:**
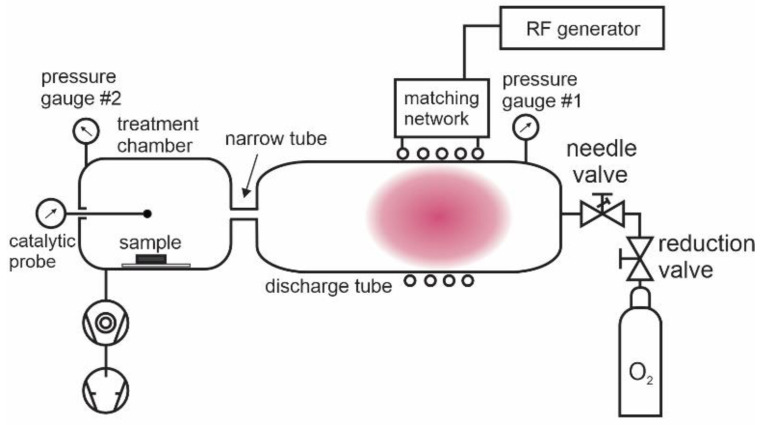
Schematic of the experimental system.

The wettability of polymer samples was studied using the experimental setup presented in Figure 2. Commercially available oxygen of purity 99.999% was introduced to the discharge tube, which was made from borosilicate glass. The pressure in the discharge tube was measured with a capacitive absolute pressure gauge (Baratron 722A, MKS Instruments, Andover, MA, USA). Plasma was sustained in the discharge tube within a water-cooled copper coil, which was connected to an RF generator via a matching network. A 13.56 MHz and 1 kW RF generator (Cesar 1210, Advanced Energy, Denver, CO, USA) was used. Plasma in the discharge tube was coupled either in the E or H mode, depending on the absorbed power. Glowing plasma occupied the entire discharge chamber when the coupling was in the E mode, but it was concentrated in the volume within the coil when the coupling was in the H mode. The illustration in Figure 2 is for the H mode. There was a narrow glass tube between the discharge chamber and the treatment chamber, where the sample was placed. The role of the narrow glass tube was to prevent the spreading of the glowing plasma into the treatment chamber. The gas in the treatment chamber was, therefore, free from short-living plasma species but rich in long-living radicals. The treatment chamber was also free from any radiation from glowing plasma. The turbomolecular and rotary pumps (HiPace 80, Pfeiffer Vacuum, Aßlar, Germany, and Trivac D16B, Leybold, Cologne, Germany, respectively) enabled the rapid evacuation of the treatment chamber. A high speed of the gas drifting from the discharge tube to the treatment chamber through the narrow tube ensured the constant supply of long-leaving plasma species into the treatment chamber where polymer samples were placed. The long-leaving oxygen species are neutral atoms in the ground state and neutral molecules in both ground and metastable excited states. Neutral oxygen atoms are stable at low-pressure conditions because a three-body collision is required for the gas-phase association, and the collision frequency is marginal at pressures used in our experiments (up to 30 pa) [4]. The radiative lifetime of oxygen molecules in the first and second metastable states (a^1^Δ and b^1^Σ) is about 45 min and 10 s, respectively, and the relaxation on the glass surfaces is highly improbable [31]. Therefore, the molecules are dense even in the late afterglow of oxygen plasma [32]. Charged particles and metastable atoms of a short lifetime neutralized and relaxed on the way from the discharge tube to the treatment chamber; therefore, they were absent in the treatment chamber. The treatment chamber was equipped with a catalytic probe (thermocouple cobalt probe, Plasmadis, Ljubljana, Slovenia) for measuring the density of neutral oxygen atoms in the ground state and a pressure gauge (PBR 260, Pfeiffer Vacuum) to measure the gas pressure. The absolute inaccuracy of the catalytic probe is around 20% in the probing range between approximately 3 × 10^18^ and 3 × 10^21^ m^−3^. Details about the probe calibration and catalytic properties of the cobalt probes have been provided elsewhere [33].

**Figure 3:**
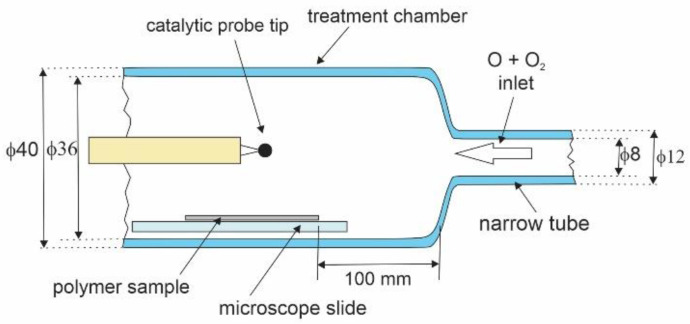
Detail of the treatment chamber with dimensions in mm. Not to scale.

The treatment chamber is shown in Figure 3. It was made from borosilicate glass and kept at room temperature. The inner diameter of the treatment chamber tube was 36 mm, and of the narrow tube, it was 8 mm. The tip of the catalytic probe was placed about 1 cm above the sample, as shown in Figure 3. The probe was movable, so we could measure the gradient of O-atom density along the treatment chamber by moving the position of the probe tip from one side to the other side of the polymer sample. The gradients were marginal as the O-atom density at the position above the sample edge facing the inlet from the narrow tube was, at most, by a factor of 1.1 higher than at the position above the sample edge facing the pump duct. The 10% is below the inaccuracy of the catalytic probe (as mentioned above, it is around 20%), so the O-atom density during the treatment of the polymer samples was measured at a fixed position of the catalytic probe tip, as shown in Figure 3.

Before starting the systematic treatment of polymer samples, we evacuated the entire experimental system for an hour to ensure the removal of trace gases from the system. Then, the system was vented with dry air, and a sample was placed into the treatment chamber at the position shown in Figure 3. The system was then evacuated again to the ultimate pressure, which was well below 0.01 Pa. Once the pressure dropped below 0.01 Pa, we opened the needle valve and left the oxygen flow for 5 min before turning on the RF generator. Plasma was on for a selected period (depending on other conditions, from 10 ms to 1000 s) to enable oxygen atoms in the treatment chamber to interact with the polymer samples. After accomplishing the polymer sample treatment, the system was vented again with dry air, the treatment chamber was opened, and the sample was probed by WCA within a minute after the treatment to minimize any influence of aging effects on the measured water contact angle. The density of O atoms in the treatment chamber was adjusted by changing the discharge power or opening the needle valve. The discharge power varied between 40 and 500 W, and the pressure in the treatment chamber, as adjusted by the needle valve, varied between 0.05 and 30 Pa. Such a broad range of the external parameters (pressure and discharge power) enabled obtaining the O-atom density in the treatment chamber between approximately 3 × 10^18^ and 3 × 10^21^ m^−3^—it was adjustable in three orders of magnitude.

### 2.3. Determination of the Wettability

The static contact angles of water droplets (WCA) were measured by the sessile drop method. We used a professional drop-shape analyzer (DSA100E, Krüss GmbH, Hamburg, Germany). A droplet of miliQ water with a volume of 1 µL was deposited onto a polymer sample. The Ellipse-Tangent fitting method was used to determine the shape of the water droplet and the corresponding contact angle. Five drops were applied to the surface of each sample to estimate the statistical error. The values of the water contact angle reported in the text below are thus averaged over five measurements. Contact angles were measured immediately (i.e., within a few minutes) after plasma treatment. Such a short time prevented significant hydrophobic recovery by the mobility of the polymer chains and/or reorientation of the surface polar functional groups. The measurable hydrophobic recovery may occur in minutes at elevated temperatures [34], but at room temperature, a measurable change in WCA was observed only after approximately half an hour of storage at ambient conditions [35].

## 3. Results and Discussion

**Figure 4:**
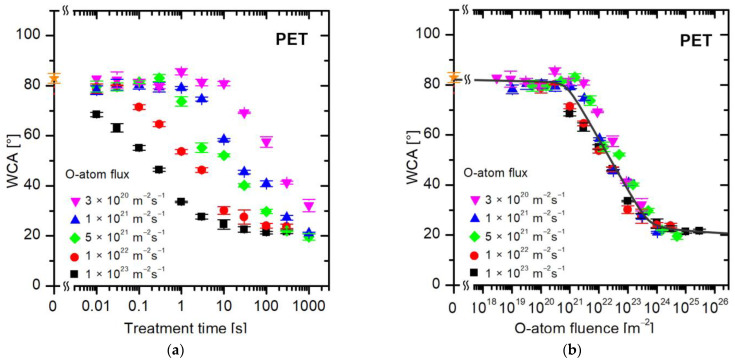
The water contact angle on the PET surface versus (**a**) the treatment time and (**b**) the fluence (dose) of oxygen atoms in the ground state. The O-atom fluxes are marked with different colors. The orange star symbol represents the WCA value of the untreated sample.

Individual samples were exposed to different fluxes of oxygen atoms. The O-atom density (*n*) in the chamber varied in a broad range between 3 × 10^18^ and 3 × 10^21^ m^−3^. The resultant flux of O atoms was calculated using the standard relation j=14nv, where *n* is the density of O atoms in the ground state as determined by the catalytic probe, and 〈*v*〉 is the average random velocity of O atoms, i.e., $\langle v\rangle =\sqrt{8kT/\pi m}$, where *k* is the Boltzmann constant, and *m* is the mass of an O atom. The velocity 〈*v*〉 is 628 m/s at room temperature. The samples were treated at a selected flux of O-atoms for various periods. Figure 4a represents the water contact angles versus the treatment time of polyethylene terephthalate (PET) samples at several selected fluxes of O atoms between 1 × 10^20^ and 1 × 10^23^ m^−2^s^−1^. The treatment time spanned between 10 ms and 1000 s, so five orders of magnitude.

The first observation in Figure 4a is that the WCA remains intact at low fluxes and reasonably large treatment times. For example, the WCA remains unchanged up to the treatment time of a second at the lowest flux of 3 × 10^20^ m^−2^s^−1^. As values of either of these variables are increased, the WCA decreases with increasing treatment time, but huge differences are observed between different fluxes of O-atoms. For example, a few seconds of treatment at a flux of 1 × 10^23^ m^−2^s^−1^ causes saturation in the wettability of PET samples (reaching a constant WCA of approximately 20°), while the hydrophobic character remains intact (the WCA is the same as for the untreated samples, i.e., approximately 80°) at a flux of 3 × 10^20^ m^−2^s^−1^. It is obvious that a certain WCA can be achieved practically at any treatment time, so the treatment time is not the parameter that governs the surface wettability.

The better parameter governing the WCA is the fluence (dose) of O atoms. Figure 4b shows exactly the same data as Figure 4a, but plotted versus the O-atom fluence instead of the treatment time. The fluence is calculated as the product of the flux and the treatment time, as long as the flux is constant over the entire treatment period. The experimental system shown in Figure 2 and Figure 3 assures a constant O-atom density at selected conditions (i.e., discharge power and opening of the needle valve). Therefore, the fluence is just a product of the flux and the treatment time in our experimental system. In Figure 4b, the WCA data measured at various fluxes overlap within the limits of the experimental error, so it is obvious that the fluence of O atoms is the parameter governing the wettability of PET polymer. We should stress again that the measured points shown in Figure 4b are exactly the same as in Figure 4a. The only difference is that in Figure 4a, the WCA is plotted versus the treatment time, and in Figure 4b, the WCA is plotted versus the fluence (dose) of oxygen atoms.

The results plotted in Figure 4b enable the following conclusions:

1. Not much in terms of increased wettability occurs on the PET surface until the O-atom dose reaches 1 × 10^21^ m^−2^. Any WCA deviation from a pristine PET is within the limits of experimental error up to the O-atom dose of 10^21^ m^−2^.
2. After initiating the surface hydrophilization at doses of approximately 1 × 10^21^ m^−2^, the WCA decreases exponentially until it reaches the minimal WCA of approximately 20°.
3. After receiving the dose of approximately 5 × 10^23^ m^−2^, the WCA remains constant at approximately 20° for another order of magnitude larger O-atom fluences. Obviously, all changes in the surface wettability of PET samples occur within the range of O-atom doses between 10^21^ and 10^24^ m^−2^. This observation is consistent with the data provided by Akishev et al. [36], who also reported stabilization of the WCA after receiving the O-atom fluence of approximately 5 × 10^23^ m^−2^ when using atmospheric-pressure plasma for a rapid increase in PET wettability.

**Figure 5:**
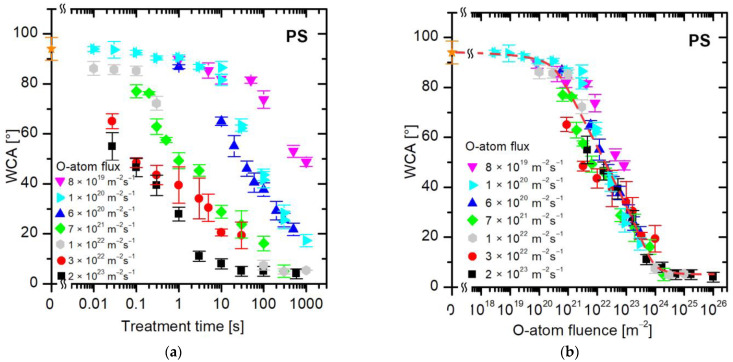
The water contact angle on the PS surface versus (**a**) the treatment time and (**b**) the fluence of oxygen atoms in the ground state. The O-atom fluxes are marked with different colors. The orange star symbol represents the WCA value of the untreated sample.

Similar results as for PET (Figure 4) were also obtained for polystyrene (PS). Figure 5 shows the evolution of the WCA for PS samples, where the WCA in Figure 5a is plotted versus the treatment time and in Figure 5b versus the dose of O atoms. The results are similar to those observed for PET, except that the initial WCA is larger and the final is smaller. In fact, the final WCA is close to the detection limit of our method, which is a few degrees. The treatment of PS by oxygen atoms at fluences larger than approximately 2 × 10^24^ m^−2^ enables an almost super-hydrophilic surface finish (Figure 5b).

**Figure 6:**
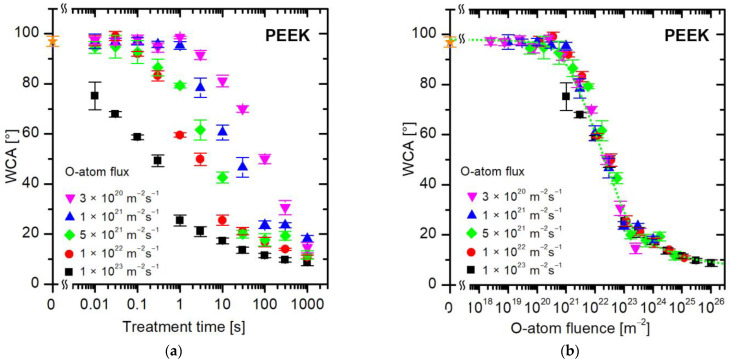
The water contact angle on the PEEK surface versus (**a**) the treatment time and (**b**) the fluence of oxygen atoms in the ground state. The O-atom fluxes are marked with different colors. The orange star symbol represents the WCA value of the untreated sample.

The evolution of the water contact angle of the PEEK polymer during treatment with oxygen atoms is shown in Figure 6. Figure 6a represents the WCA versus the treatment time. As for PET (Figure 4a) and PS (Figure 5a), no measurable deviation in the WCA from the value typical for untreated samples (approximately 97°) occurs at short treatment times or very low O-atom fluxes. Figure 6b represents the WCA versus the fluence of O atoms. The hydrophobicity (high WCA above 90°) remains intact at low fluences of O atoms up to a few 10^20^ m^−2^. Moderate fluences in the range from approximately 10^21^ to 10^23^ enable an almost exponential decrease in the WCA, and large fluences cause a marginal but yet measurable decrease in the WCA. Therefore, the treatment of PEEK by oxygen atoms at large fluences enables very low WCA, but the super-hydrophilic surface finish is not observed up to the O-atom fluence of 1 × 10^26^ m^−3^. Here, it is worth mentioning that Botel et al. [37] reported immeasurably low WCA on PEEK samples after treating them with capacitively coupled oxygen plasma for a few minutes, but the selected samples were rough because they were sand-blasted before the plasma treatment. Other authors reported rather inadequate wettability of this polymer after treatment with oxygen plasma, indicating that a WCA of approximately 10° is not trivial to achieve when treating PEEK in the glowing plasma [38].

**Figure 7:**
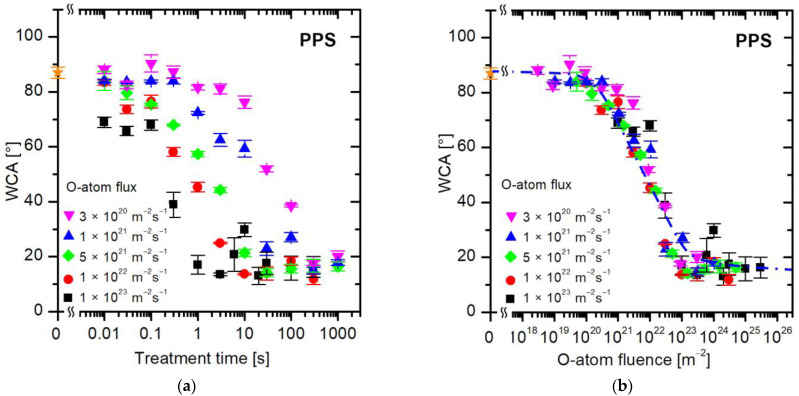
The water contact angle on the PPS surface versus (**a**) the treatment time and (**b**) the fluence of oxygen atoms in the ground state. The O-atom fluxes are marked with different colors. The orange star symbol represents the WCA value of the untreated sample.

Figure 7 shows the evolution of wettability for a sulfur-containing polymer, PPS. Again, the values of WCA versus the treatment time (Figure 7a) are scattered, so the treatment time does not tell much about the surface finish. Figure 7b is a plot of measured WCA versus the fluence of oxygen atoms. The curve in Figure 7b is similar for other polymers (Figure 4b, Figure 5b and Figure 6b), so the evolution of surface wettability of this aromatic polymer containing sulfur follows the same trend: the WCA remains intact up to the O-atom fluence of approximately 10^20^ m^−2^, decreases rapidly in the range between 10^21^ and 10^23^ m^−2^, and stabilizes thereafter.

**Figure 8:**
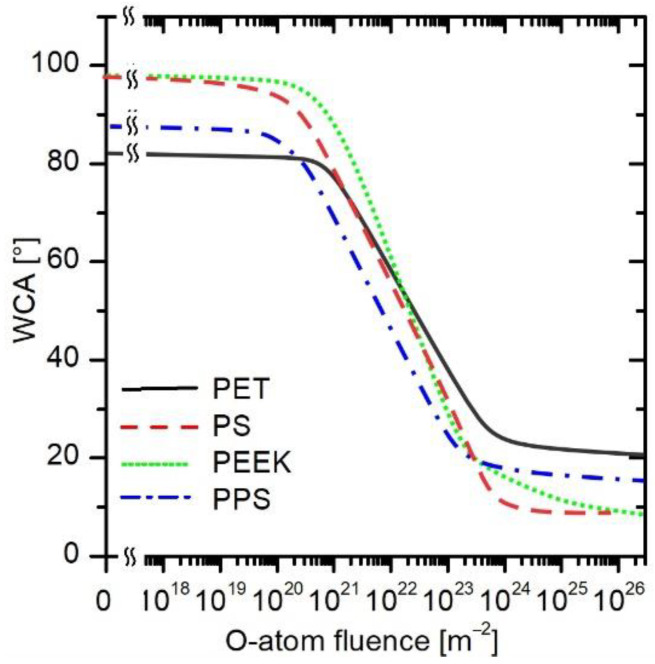
The evolution of water contact angles for PET, PS, PEEK, and PPS versus the fluence measured at various fluxes of O-atoms.

The general trend revealed in Figure 4b, Figure 5b, Figure 6b and Figure 7b seems similar, but there are small differences that are statistically relevant. The best fit for all measured WCAs (irrespective of the O-atom flux) for all four polymers is plotted in Figure 8 and represented by solid, dashed, or dotted curves. The statistically significant variation in the wettability of untreated polymer samples, i.e., the decrease in the WCA by more than 10% of the original value, is observed for all four polymers at a fluence of approximately 10^21^ m^−2^. Before receiving this fluence, nothing significant happened on the polymer surface as long as WCA was the merit.

At a fluence of approximately 10^23^ m^−2^, a WCA of 25° is observed for all aromatic polymers. This is roughly the minimal achievable water contact angle on the surface of smooth polymers [39]. At even higher fluences, there are some differences between polymers, but only about a few degrees. At a fluence of 10^26^ m^−2^, all polymers have a WCA of approximately 15 ± 5°.

Between the O-atom fluence of 5 × 10^20^ and 5 × 10^23^ m^−2^, the WCA decreases fairly linearly in Figure 8. Considering the logarithmic scale on the *x*-axis, the general curve deduced from Figure 8 follows the exponential decrease in the WCA. The exponential decrease is typical for approaching a saturated state of matter. In the case of wettability, the saturation is attributed to the formation of a layer of polar functional groups on the polymer surface [40].

**Figure 9:**
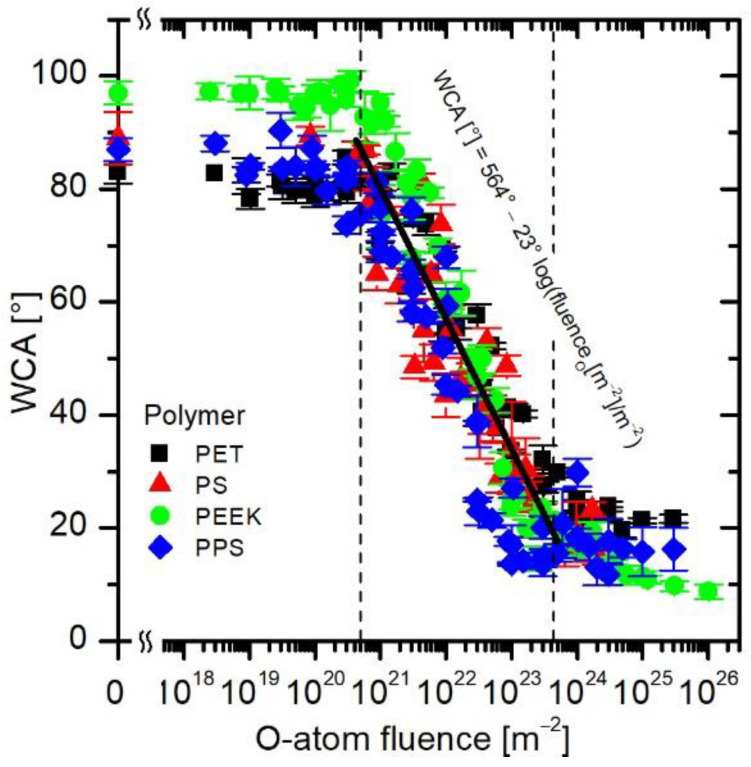
Similar behavior of all tested polymers enables obtaining a general expression for the dependence of the wettability on O-atom fluence.

Last but not least, because the curves in Figure 8 are similar and have the same trend, they allow obtaining a more general relation between a desired wettability and O-atom fluence. Therefore, all measured points from Figure 4b, Figure 5b, Figure 6b and Figure 7b were joined in Figure 9, and the corresponding equation was obtained by fitting all the measured points. The equation is displayed in Figure 9 and is valid in the range between approximately 5 × 10^20^ and 5 × 10^23^ m^−2^. From the empirical formula for calculating WCA from the O-atom fluence, one can see that by increasing the fluence by one order of magnitude, the WCA is decreased by approximately 23°. The obtained equation also enables a user to choose the right treatment parameters regardless of the configuration of the plasma device, as long as the O-atom density is known and no other plasma species are present.

A detailed description of the mechanisms involved in a polymer surface modification upon exposure to O-atoms is yet to be developed. The closest state-of-the-art in aromatic polymers is the paper by Longo et al. [41]. Longo identified over 10 adsorption sites for O atoms on a pristine PS surface, and the substitution of hydrogen on the aromatic ring with the OH group was found to be energetically most favorable. According to this theory, the formation of OH groups on the PS surface should occur preferentially. Unfortunately, Longo et al. [41] did not report the evolution of surface functional groups versus the fluence of O atoms but rather versus the surface occupancy. Contrary to Longo et al., Kushner’s group studied the evolution of functional groups on a PS surface versus the fluence of O atoms and found a significant concentration of OH groups already below the fluence of 10^20^ m^−3^ [42]. In fact, Polito et al. [42] reported saturation of the PS surface with polar functional groups already at the O-atom fluence of approximately 10^21^ O-atoms per m^2^. Polito et al. [42], however, did not take into account etching and, thus, degradation of the aromatic rings upon large fluences of O-atoms. Still, Kushner’s theory [42] predicts saturation at the fluence of 10^21^ m^−2^, while results summarized in Figure 6 clearly show that the saturation (i.e., stable low WCA) occurs at approximately 100 times larger fluence. The discrepancy could be explained by the non-trivial relation between the surface functional groups and the wettability or by the influence of the surface morphology.

The only experimental results on the evolution of specific surface functional groups on PS versus the fluence of O atoms were reported by Vesel et al. [43]. Some experiments were also performed in a plasma reactor attached to the X-ray photoelectron spectroscopy (XPS) chamber, so the evolution of functional groups was monitored without breaking the vacuum conditions [44]. These experiments are sound with the qualitative predictions of Longo et al. [41] and with the quantitative predictions provided by Polito et al. [42]. A feasible explanation for the discrepancy between the formation of oxygen functional groups on the PS surface [41,43] and the results represented in Figure 8 is the orientation of oxygen-containing functional groups on the polymer surface. A good wettability (a low WCA) is a consequence of the polar groups right on the surface. If the oxygen-rich groups are incorporated into the subsurface layer, they will not contribute to wettability. The initial interaction between oxygen atoms and the polymer definitely forms OH groups on the surface, as shown in our previous papers [43,44] and by Kushner’s group [42]. According to both theoretical [41,42] and experimental [43,44] results, the OH groups will saturate the polymer surface already at a low O-atom fluence of the order of 10^21^ m^−2^. The OH groups, however, may reorient toward a thermodynamically more stable state, i.e., away from the surface. If the reorientation happens, the OH groups will not contribute to the surface wettability. Reorientation is the most frequently mentioned channel for the hydrophobic recovery of polymers [45].

The effect may be suppressed when a somehow thicker oxygen-rich surface film is obtained and/or when the polymer surface is functionalized with other oxygen groups (e.g., containing double bonds and highly oxidized groups). For example, Kim et al. [46] treated a polymer at an elevated temperature of 100 °C with a capacitively coupled RF oxygen plasma to obtain a thicker oxide film than when treating the samples at room temperature. The oxygen concentration, as measured by XPS, was large for the sample treated at 100 °C, but the wettability was poor. The team cooled the sample and treated it again at room temperature to obtain good wettability. The hydrophobic recovery was suppressed dramatically using this two-step processing as compared to samples treated with oxygen plasma only once at room temperature.

The formation of highly oxidized groups requires much larger doses of O-atoms and also partial degradation of the aromatic ring [41,43,44]. Unfortunately, Kushner’s group did not provide a significant concentration of double bonds even after larger O-atom doses, but the experimental results [43,44] indicate a significant amount of carbonyl, carboxyl, ester, etc. groups only after treatment with the O-atom fluences in the range of 10^22^–10^23^ m^−2^. Once again, we have to stress that Polito et al. [42] did not take into account the degradation of the aromatic ring, which Longo et al. [41] found crucial for the formation of functional groups other than hydroxyl.

Finally, it is worth mentioning that the oxidation of polymer surfaces with oxygen atoms often causes polymer chain scission and the formation of low-molecular-weight highly oxidized fragments (LMWOMs) [47]. It was reported that agglomerates of such highly oxidized fragments can be formed on polymers when overtreated. Their formation depends on the polymer type and strongly on treatment conditions. The formation of LMWOMs was extensively investigated for PP polymers treated in a corona discharge, flame, or DBD; however, there are not many publications on other polymers [48,49,50,51]. Agglomerates of such fragments were reported for plasma-treated PEEK, PET, and PES [52,53] but not for PS treated at the same conditions [52]. It was reported that UV/VUV radiation and O and OH species are responsible for their formation [48,49]. At our experimental conditions, the samples were exposed only to O atoms, so their formation should be to a lesser extent. The kinetics of the formation of LMWOMs are thus yet to be elaborated, but their existence will definitely influence surface wettability. Nevertheless, it is also worth mentioning our previous papers, where we have investigated the functionalization of PS versus the O-atom dose by XPS [19,43,44] and etching effects by using a quartz crystal microbalance (QCM) and atomic force microscopy (AFM) analysis [54].

## 4. Conclusions

A carefully designed experimental setup enabled a quantitative study of the evolution of surface wettability versus the fluence of neutral oxygen atoms in the ground electronic state. The flexibility of the experimental setup enabled variation in the O-atom flux on the polymer samples from approximately 10^20^ to 10^23^ m^−2^s^−1^. We probed four frequently used aromatic polymers: polyethylene terephthalate (PET), polystyrene (PS), polyether ether ketone (PEEK), and polyphenylene sulfide (PPS). Despite the differences in the chemical structure and oxygen concentration of the pristine polymers, surface wettability followed a very similar evolution for all aromatic polymers. No decrease in the static water contact angle was observed up to the O-atom fluence of 10^20^ m^−2^. A statistically significant deviation from the value typical for non-treated samples was observed in the range of fluences between 10^20^ and 10^21^ m^−2^. Thereafter, an exponential decrease in the WCA was observed up to the fluence of approximately 10^23^ m^−2^. Larger fluences did not cause a significant decrease in the WCA. The fluences up to approximately 10^26^ m^−2^ were probed. Such a large fluence corresponded to the treatment in the afterglow chamber of the experimental system, where the plasma was sustained for 1000 s at a pressure of 20 Pa and a discharge power of 500 W. Smaller fluences were achieved at shorter treatment times and/or discharge powers. We varied the treatment time between 10 ms and 1000 s and the pressure in the tube where the samples were processed with O-atoms from 0.05 Pa to 3 Pa. A comparison of the results reported in this manuscript with the available literature indicates a non-trivial relationship between the wettability as determined by the static water droplet contact angle and the concentration of oxygen-containing surface functional groups as probed by surface-sensitive techniques like X-ray photoelectron spectroscopy.

## Figures and Tables

**Table 1:** A summary of different wettability obtained by authors in various experimental conditions using oxygen plasma treatment.

Reference	Polymer	Plasma Configuration	Power or Power Density	Pressure or Flow	WCA Initial	WCA Final	Time
[12]	PET	CCP-RF	75 W	1.3 Pa	72°	<5°	10 min
[13]	PET	ICP-RF diffusing afterglow	400 W	0.02 Pa	75°	~20°	24 min
[14]	PET	CCP RF	20–100 W	5–100 Pa	/	~0°	Order of minutes
[15]	PET	CCP-RF	180 W	/	93°	34°	Order of minutes
[16]	PET	APJ	Few W	Atmospheric Ar	80°	<25°	20 s
[17]	PS	ERC MW Electron Cyclotron Resonance	100–200 W	0.1 Pa	66°	46° at 200 W	3 min
[18]	PS	ICP RF	10 W	66 Pa	88°	5°	210 s
[19]	PS	ICP RF	200 W	75 Pa	86°	3°	20 s
[20]	PS	CCP RF	200 W	15 sccm	71°	8°	Order of minutes
[21]	PS	CCP RF	0.18 W	Ar with oxygen impurities	91°	20°	180 s
[22]	PEEK	CCP RF glowing part	20–60 W			17° at 20 W 10° at 60 W	10 s
[22]	PEEK	CCP RF afterglow	20–60 W			25° or 22° *	30 s
[23]	PEEK	Plasma ion implantation	100–150 W	63–66 Pa	~90°	~40°	256 s
[24]	PEEK	RF CCP	100 W	100 Pa	~90°	25° or 38° **	15 min
[25]	PEEK	UV-induced O_2_ plasma	/	/	74°	64°	20 s
[26]	PEEK	RF CCP	/	/	80°	~0°	10 min
[27]	PPS	Atmospheric RF	120 W	Atmospheric Ar/O_2_	96°	3°	120 s
[28]	PPS		400–1200 W	100, 200 sccm	85°	~40–50°	10–600 s
[29]	PPS	Atmospheric DBD	5.3 W/cm^3^	Atmospheric air	111°	~30°	15 s
[30]	PPS	Atmospheric DBD	22 W/cm^3^	Atmospheric air	79°	40°	6 s

* for 20 or 60 W, respectively; ** for deposited or polished PEEK, respectively.

## Data Availability

All data are contained within the article.
